# 904 Impact of Socioeconomic and Psychological Determinants on the Incidence and Outcomes of Pediatric Burn Injuries

**DOI:** 10.1093/jbcr/iraf019.435

**Published:** 2025-04-01

**Authors:** Aishwarya Raj, Maneesh Singhal, Shivangi Saha

**Affiliations:** AIIMS, New Delhi India; AIIMS, New Delhi India; AIIMS, New Delhi India

## Abstract

**Introduction:**

Burn injuries impact can be Socioeconomic factors, including parental income, education, and housing conditions, significantly influence the risk of burn injuries. Additionally, psychological determinants such as parental stress and caregiver mental health can impact both the incidence of burns and recovery outcomes.

**Methods:**

This epidemiological study spanned one year and involved 68 pediatric patients aged 0-18 admitted for acute burn injuries at the department. Data was collected from patients and their families to assess the influence of socioeconomic and psychological factors on burn incidence and outcomes.

2. After following inclusion and exclusion criteria Clinical Data was collected on: demographics, burn type and extent (total body surface area and depth), cause (e.g., scalds, flame, electrical), and clinical management details, including treatment and recovery outcomes. Socioeconomic Data: Structured interviews with parents on income, education, employment status, housing conditions, and home safety measures (e.g., fire extinguishers, first aid kits, elevated cooking space). Psychological Data on Parental Mental Health by the General Health Questionnaire (GHQ) for anxiety, depression, and stress, Caregiver Burden Inventory (CBI), Family APGAR Scale to evaluate support and cohesion in coping with the child’s injury. Appropriate statistical methods were used to assess the data.

**Results:**

Preliminary findings from the study involving 68 pediatric burn patients indicate strong associations between socioeconomic status and injury severity. Children from lower-income families experienced more extensive burns and longer hospital stays. Parental mental health issues, such as elevated anxiety and depression, were also linked to challenges in recovery outcomes. Additionally, caregivers reported high emotional burden, especially in cases of prolonged treatment and complications. Further analysis is currently underway to deepen our understanding of these relationships and their implications for future interventions.

**Conclusions:**

The study highlights how socioeconomic and psychological factors play a critical role in determining the incidence and outcomes of pediatric burn injuries in developing countries. Lower-income status and heightened caregiver stress were linked to more severe injuries and poorer recovery outcomes.

**Applicability of Research to Practice:**

The applicability of this study lies in its potential to create real-world impact by addressing the unique challenges faced by children and families dealing with burn injuries in developing countries. By examining both socioeconomic and psychological factors, this research goes beyond clinical data to highlight the underlying vulnerabilities that increase burn risks and impede recovery. The findings can directly inform healthcare policies, guiding the development of prevention programs and resource allocation to communities most in need.

**Funding for the Study:**

N/A

